# *p*-Tolyl bis­(*o*-tolyl­amido)­phosphinate

**DOI:** 10.1107/S1600536810023512

**Published:** 2010-06-23

**Authors:** Fahimeh Sabbaghi, Teresa Mancilla Percino, Mehrdad Pourayoubi, Marco A. Leyva

**Affiliations:** a Department of Chemistry, Islamic Azad University-Zanjan Branch, PO Box 49195-467, Zanjan, Iran; b Departamento de Química, Centro de Investigación y de Estudios Avanzados, del Instituto Politécnico Nacional, Apartado Postal 14-740, 07000 México, D.F., México; c Department of Chemistry, Ferdowsi University of Mashhad, 91779, Mashhad, Iran

## Abstract

In the title compound, C_21_H_23_N_2_O_2_P, the P atom has a distorted tetra­hedral configuration. The O atom of the OC_6_H_4_-4-CH_3_ group and the N atoms show *sp* ^2^ character. In the crystal, adjacent mol­ecules are linked by N—H⋯O hydrogen bonds into helical chains parallel to the *b* axis.

## Related literature

For a related structure, see: Pourayoubi *et al.* (2009 ▶).
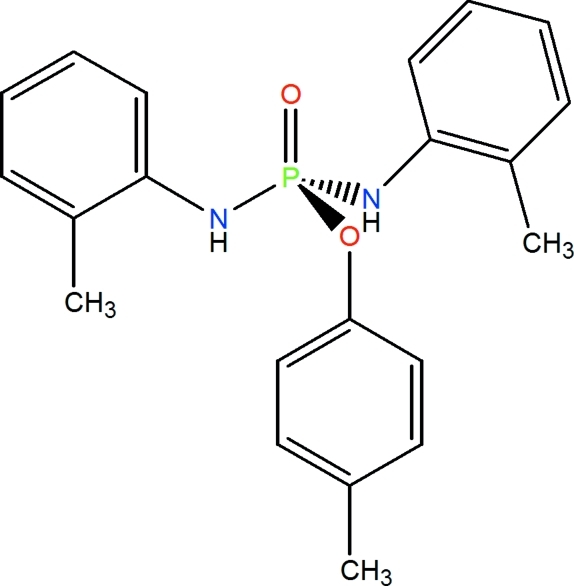


## Experimental

### 

#### Crystal data

C_21_H_23_N_2_O_2_P
*M* *_r_* = 366.38
Monoclinic, 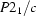

*a* = 12.157 (3) Å
*b* = 8.978 (2) Å
*c* = 18.080 (5) Å
β = 101.569 (1)°
*V* = 1933.3 (8) Å^3^
*Z* = 4
Mo *K*α radiation
μ = 0.16 mm^−1^
*T* = 293 K
0.6 × 0.54 × 0.47 mm

#### Data collection

Nonius KappaCCD diffractometer
Absorption correction: multi-scan (Blessing, 1995 ▶) *T*_min_ = 0.860, *T*_max_ = 0.968
23372 measured reflections
4402 independent reflections
3097 reflections with *I* > 2σ(*I*)
*R*_int_ = 0.048

#### Refinement

*R*[*F* ^2^ > 2σ(*F* ^2^)] = 0.045
*wR*(*F* ^2^) = 0.128
*S* = 1.06
4402 reflections
257 parameters
2 restraints
H atoms treated by a mixture of independent and constrained refinement
Δρ_max_ = 0.18 e Å^−3^
Δρ_min_ = −0.36 e Å^−3^

### 

Data collection: *COLLECT* (Nonius, 2001 ▶); cell refinement: *HKL* *SCALEPACK* (Otwinowski & Minor, 1997 ▶); data reduction: *HKL* *DENZO* (Otwinowski & Minor, 1997 ▶) and *SCALEPACK*; program(s) used to solve structure: *SHELXS97* (Sheldrick, 2008 ▶); program(s) used to refine structure: *SHELXL97* (Sheldrick, 2008 ▶); molecular graphics: *Mercury* (Macrae *et al.*, 2006 ▶); software used to prepare material for publication: *WinGX* (Farrugia, 1999 ▶).

## Supplementary Material

Crystal structure: contains datablocks I, global. DOI: 10.1107/S1600536810023512/ng2779sup1.cif

Structure factors: contains datablocks I. DOI: 10.1107/S1600536810023512/ng2779Isup2.hkl

Additional supplementary materials:  crystallographic information; 3D view; checkCIF report

## Figures and Tables

**Table 1:** Hydrogen-bond geometry (Å, °)

*D*—H⋯*A*	*D*—H	H⋯*A*	*D*⋯*A*	*D*—H⋯*A*
N1—H1⋯O1^i^	0.91 (2)	2.02 (2)	2.8963 (19)	161 (2)

Symmetry code: (i) 
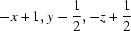
.

**Fig. 1.**
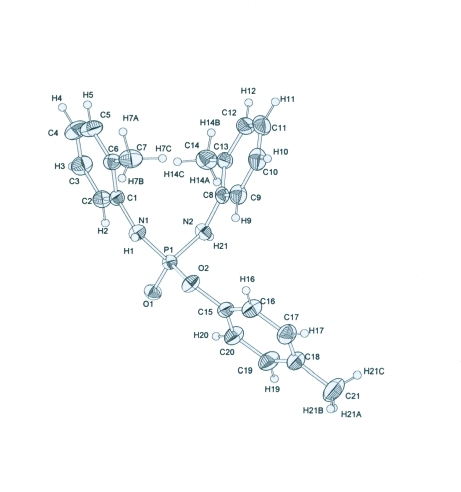
A general view of the title compound, showing the atom-numbering scheme. Displacement ellipsoids are drawn at the 50% probability level.

**Fig. 2.**
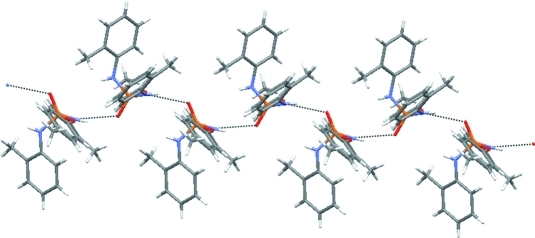
A view of N—H···O hydrogen bond.

## supplementary crystallographic information

### Comment

In the previous work, the structure determination of *p*-tolyl bis(*p*-tolylamido)phosphate (Pourayoubi *et al.*, 2009) has been investigated; we report here on the crystal structure of title compound (Fig. 1). The title compound was synthesized from the reaction of (4-tolyl)-dichlorophosphate with an excess amount of *ortho*-toluidine (1:4 mole ratio). Single crystals were obtained from CHCl_3_/n-C_6_H_14_ at room temperature. Molecular structure of [4-H_3_C—C_6_H_4_O]P(O)[NHC_6_H_4_-2-CH_3_]_2_ is shown in Fig. 1. The phosphorus atom has a distorted tetrahedral configuration. The bond angles around P atom are in the range of 96.87 (7)° to 118.95 (8)°. The oxygen atom of OC_6_H_4_-4-CH_3_ moiety and the nitrogen atoms show *sp*^2^ character (the C15—O2—P1 angle is 124.67 (11)°, the C1—N1—P1 and C8—N2—P1 are 123.77 (12)° and 127.71 (12)°, respectively. In the crystal structure, molecules are linked *via* N—H···O hydrogen bonds (N1···O1 = 2.8963 (19) Å) into an extended chain (Fig. 2) parallel to the *b* axis.

### Experimental

To a solution of (4-tolyl)-dichlorophosphate (2.250 g, 10 mmol) in 15 ml dry acetonitrile, a solution of *ortho*-toluidine (4.286 g, 40 mmol) in 30 ml acetonitrile was added at 0°C. After 4 h stirring, the solvent was evaporated in vacuum. The solid was washed with distilled water. Single crystals of the product were obtained from a solution of CHCl_3_/n-C_6_H_14_ at room temperature.

### Refinement

H atoms of both nitrogen were found by Fourier differences, it was necessary to restrain distances setting the NH as 1.01 Å instead of 0.86 Å as the ideal would be, but under this proposal to refine, both distances are obtained, 0.9119 (152) Å for N1—H1 and 0.8982 (153) Å for N2—H21,respectively, which are more realistic. The difference can be due to the effect of hydrogen bond generates by N1—H1—O1. All other hydrogen atoms were placed geometrically.

### Crystal data

**Table:**

C_21_H_23_N_2_O_2_P	*F*(000) = 776
*M**_r_* = 366.38	*D*_x_ = 1.259 Mg m^−^^3^
Monoclinic, *P*2_1_/*c*	Mo *K*α radiation, λ = 0.71073 Å
Hall symbol: -P 2ybc	Cell parameters from 600 reflections
*a* = 12.157 (3) Å	θ = 1–14°
*b* = 8.978 (2) Å	µ = 0.16 mm^−^^1^
*c* = 18.080 (5) Å	*T* = 293 K
β = 101.569 (1)°	Priem, colourless
*V* = 1933.3 (8) Å^3^	0.6 × 0.54 × 0.47 mm
*Z* = 4	

### Data collection

**Table:**

Nonius KappaCCD diffractometer	4402 independent reflections
Radiation source: Enraf Nonius FR590	3097 reflections with *I* > 2σ(*I*)
graphite	*R*_int_ = 0.048
Detector resolution: 9 pixels mm^-1^	θ_max_ = 27.5°, θ_min_ = 2.6°
CCD rotation images, thick slices scans	*h* = −15→15
Absorption correction: multi-scan (Blessing, 1995)	*k* = −10→11
*T*_min_ = 0.860, *T*_max_ = 0.968	*l* = −19→23
23372 measured reflections	

### Refinement

**Table:**

Refinement on *F*^2^	Primary atom site location: structure-invariant direct methods
Least-squares matrix: full	Secondary atom site location: difference Fourier map
*R*[*F*^2^ > 2σ(*F*^2^)] = 0.045	Hydrogen site location: inferred from neighbouring sites
*wR*(*F*^2^) = 0.128	H atoms treated by a mixture of independent and constrained refinement
*S* = 1.06	*w* = 1/[σ^2^(*F*_o_^2^) + (0.0666*P*)^2^ + 0.2653*P*] where *P* = (*F*_o_^2^ + 2*F*_c_^2^)/3
4402 reflections	(Δ/σ)_max_ = 0.015
257 parameters	Δρ_max_ = 0.18 e Å^−^^3^
2 restraints	Δρ_min_ = −0.36 e Å^−^^3^

### Special details

**Table:**

Geometry. All s.u.'s (except the s.u. in the dihedral angle between two l.s. planes) are estimated using the full covariance matrix. The cell s.u.'s are taken into account individually in the estimation of s.u.'s in distances, angles and torsion angles; correlations between s.u.'s in cell parameters are only used when they are defined by crystal symmetry. An approximate (isotropic) treatment of cell s.u.'s is used for estimating s.u.'s involving l.s. planes.
Refinement. Refinement of *F*^2^ against ALL reflections. The weighted *R*-factor *wR* and goodness of fit *S* are based on *F*^2^, conventional *R*-factors *R* are based on *F*, with *F* set to zero for negative *F*^2^. The threshold expression of *F*^2^ > 2σ(*F*^2^) is used only for calculating *R*-factors(gt) *etc*. and is not relevant to the choice of reflections for refinement. *R*-factors based on *F*^2^ are statistically about twice as large as those based on *F*, and *R*- factors based on ALL data will be even larger.

### Fractional atomic coordinates and isotropic or equivalent isotropic displacement parameters (Å^2^)

**Table:**

	*x*	*y*	*z*	*U*_iso_*/*U*_eq_	
C1	0.28763 (13)	0.18689 (18)	0.26516 (10)	0.0359 (4)	
C2	0.25191 (16)	0.3029 (2)	0.21582 (11)	0.0464 (4)	
H2	0.3036	0.3535	0.1936	0.056*	
C3	0.14023 (18)	0.3444 (3)	0.19930 (13)	0.0606 (6)	
H3	0.1171	0.4232	0.1665	0.073*	
C4	0.06346 (18)	0.2691 (3)	0.23138 (15)	0.0693 (7)	
H4	−0.0121	0.2951	0.2197	0.083*	
C5	0.09918 (17)	0.1546 (3)	0.28107 (14)	0.0620 (6)	
H5	0.0466	0.1044	0.3027	0.074*	
C6	0.21099 (15)	0.1116 (2)	0.29999 (11)	0.0446 (4)	
C7	0.24895 (19)	−0.0078 (2)	0.35748 (13)	0.0628 (6)	
H7A	0.1847	−0.0521	0.3722	0.094*	
H7B	0.2969	0.0351	0.401	0.094*	
H7C	0.2896	−0.0827	0.3361	0.094*	
C8	0.43487 (15)	0.36933 (19)	0.41804 (9)	0.0395 (4)	
C9	0.47389 (18)	0.2563 (2)	0.46833 (11)	0.0518 (5)	
H9	0.5289	0.1915	0.4585	0.062*	
C10	0.4311 (2)	0.2392 (3)	0.53349 (12)	0.0656 (6)	
H10	0.4559	0.1615	0.5666	0.079*	
C11	0.3524 (2)	0.3374 (3)	0.54873 (13)	0.0687 (7)	
H11	0.3252	0.3283	0.5931	0.082*	
C12	0.31363 (18)	0.4494 (3)	0.49840 (12)	0.0614 (6)	
H12	0.2603	0.5155	0.5095	0.074*	
C13	0.35181 (15)	0.4668 (2)	0.43141 (10)	0.0464 (5)	
C14	0.3050 (2)	0.5866 (3)	0.37677 (16)	0.0643 (6)	
C15	0.71094 (14)	0.1945 (2)	0.36813 (10)	0.0399 (4)	
C16	0.76182 (16)	0.1141 (2)	0.43050 (12)	0.0530 (5)	
H16	0.7234	0.0378	0.4492	0.064*	
C17	0.87118 (17)	0.1486 (3)	0.46506 (13)	0.0607 (6)	
H17	0.9056	0.0944	0.5072	0.073*	
C18	0.93031 (17)	0.2605 (3)	0.43883 (13)	0.0575 (5)	
C19	0.87634 (17)	0.3392 (3)	0.37669 (13)	0.0633 (6)	
H19	0.9143	0.4163	0.3583	0.076*	
C20	0.76747 (16)	0.3071 (2)	0.34082 (12)	0.0539 (5)	
H20	0.7331	0.3613	0.2987	0.065*	
C21	1.0505 (2)	0.2947 (4)	0.47617 (17)	0.0897 (9)	
H21A	1.0534	0.3242	0.5276	0.135*	
H21B	1.0784	0.3741	0.4495	0.135*	
H21C	1.0959	0.2075	0.4751	0.135*	
N1	0.40296 (12)	0.14475 (16)	0.28185 (9)	0.0387 (3)	
N2	0.48102 (13)	0.38993 (16)	0.35183 (8)	0.0393 (3)	
O1	0.53434 (10)	0.33484 (13)	0.22624 (7)	0.0420 (3)	
O2	0.60148 (10)	0.15191 (13)	0.33463 (7)	0.0444 (3)	
P1	0.50634 (4)	0.26305 (4)	0.29312 (2)	0.03385 (15)	
H1	0.4230 (17)	0.0477 (18)	0.2912 (12)	0.062 (6)*	
H21	0.4928 (17)	0.4862 (18)	0.3421 (12)	0.061 (6)*	
H14C	0.267 (3)	0.548 (3)	0.3309 (19)	0.104 (10)*	
H14B	0.249 (3)	0.645 (4)	0.3955 (18)	0.123 (11)*	
H14A	0.363 (3)	0.660 (3)	0.3641 (17)	0.105 (10)*	

### Atomic displacement parameters (Å^2^)

**Table:**

	*U*^11^	*U*^22^	*U*^33^	*U*^12^	*U*^13^	*U*^23^
C1	0.0343 (9)	0.0320 (9)	0.0410 (9)	−0.0007 (7)	0.0063 (7)	−0.0066 (7)
C2	0.0451 (11)	0.0446 (10)	0.0496 (11)	0.0036 (8)	0.0099 (9)	0.0016 (8)
C3	0.0508 (12)	0.0606 (13)	0.0673 (13)	0.0155 (10)	0.0046 (10)	0.0094 (11)
C4	0.0381 (11)	0.0708 (15)	0.0969 (19)	0.0124 (10)	0.0085 (12)	0.0035 (13)
C5	0.0429 (12)	0.0608 (13)	0.0875 (16)	−0.0049 (10)	0.0255 (11)	−0.0025 (12)
C6	0.0434 (10)	0.0400 (10)	0.0524 (11)	−0.0032 (8)	0.0142 (8)	−0.0028 (8)
C7	0.0634 (13)	0.0583 (13)	0.0724 (14)	−0.0046 (11)	0.0272 (11)	0.0159 (11)
C8	0.0440 (10)	0.0391 (9)	0.0351 (9)	−0.0131 (7)	0.0074 (7)	−0.0069 (7)
C9	0.0580 (12)	0.0516 (12)	0.0446 (11)	−0.0084 (9)	0.0072 (9)	0.0031 (9)
C10	0.0774 (16)	0.0723 (15)	0.0447 (12)	−0.0274 (13)	0.0066 (11)	0.0083 (10)
C11	0.0781 (16)	0.0866 (17)	0.0469 (12)	−0.0379 (14)	0.0255 (11)	−0.0122 (12)
C12	0.0569 (12)	0.0702 (15)	0.0627 (13)	−0.0206 (11)	0.0254 (10)	−0.0215 (12)
C13	0.0456 (10)	0.0465 (11)	0.0488 (10)	−0.0140 (8)	0.0137 (8)	−0.0136 (8)
C14	0.0639 (15)	0.0566 (14)	0.0739 (16)	0.0124 (12)	0.0176 (13)	−0.0040 (12)
C15	0.0335 (9)	0.0392 (10)	0.0470 (10)	0.0002 (7)	0.0080 (8)	−0.0031 (8)
C16	0.0428 (11)	0.0542 (12)	0.0610 (12)	0.0002 (9)	0.0085 (9)	0.0113 (10)
C17	0.0464 (12)	0.0712 (14)	0.0597 (13)	0.0073 (10)	−0.0009 (10)	0.0060 (11)
C18	0.0394 (11)	0.0678 (14)	0.0626 (13)	−0.0017 (9)	0.0039 (10)	−0.0080 (11)
C19	0.0461 (12)	0.0682 (14)	0.0757 (15)	−0.0163 (10)	0.0125 (11)	0.0062 (12)
C20	0.0420 (11)	0.0583 (12)	0.0592 (12)	−0.0062 (9)	0.0052 (9)	0.0133 (10)
C21	0.0464 (14)	0.108 (2)	0.105 (2)	−0.0113 (14)	−0.0079 (14)	−0.0065 (18)
N1	0.0352 (8)	0.0277 (7)	0.0527 (9)	0.0005 (6)	0.0075 (6)	−0.0013 (6)
N2	0.0519 (9)	0.0282 (8)	0.0399 (8)	−0.0047 (6)	0.0144 (7)	−0.0016 (6)
O1	0.0477 (7)	0.0385 (7)	0.0428 (7)	−0.0021 (5)	0.0159 (5)	0.0007 (5)
O2	0.0335 (6)	0.0342 (6)	0.0627 (8)	−0.0016 (5)	0.0025 (6)	0.0045 (6)
P1	0.0341 (2)	0.0287 (2)	0.0391 (3)	−0.00105 (17)	0.00834 (18)	−0.00115 (17)

### Geometric parameters (Å, °)

**Table:**

C1—C2	1.383 (3)	C13—C14	1.494 (3)
C1—C6	1.399 (2)	C14—H14C	0.93 (3)
C1—N1	1.425 (2)	C14—H14B	0.97 (3)
C2—C3	1.381 (3)	C14—H14A	1.02 (3)
C2—H2	0.93	C15—C20	1.369 (3)
C3—C4	1.372 (3)	C15—C16	1.377 (3)
C3—H3	0.93	C15—O2	1.400 (2)
C4—C5	1.377 (3)	C16—C17	1.386 (3)
C4—H4	0.93	C16—H16	0.93
C5—C6	1.388 (3)	C17—C18	1.374 (3)
C5—H5	0.93	C17—H17	0.93
C6—C7	1.500 (3)	C18—C19	1.377 (3)
C7—H7A	0.96	C18—C21	1.513 (3)
C7—H7B	0.96	C19—C20	1.382 (3)
C7—H7C	0.96	C19—H19	0.93
C8—C9	1.382 (3)	C20—H20	0.93
C8—C13	1.393 (3)	C21—H21A	0.96
C8—N2	1.432 (2)	C21—H21B	0.96
C9—C10	1.388 (3)	C21—H21C	0.96
C9—H9	0.93	N1—P1	1.6268 (15)
C10—C11	1.369 (4)	N1—H1	0.911 (15)
C10—H10	0.93	N2—P1	1.6279 (15)
C11—C12	1.375 (3)	N2—H21	0.899 (15)
C11—H11	0.93	O1—P1	1.4692 (12)
C12—C13	1.390 (3)	O2—P1	1.5964 (13)
C12—H12	0.93		
			
C2—C1—C6	120.19 (16)	C13—C14—H14B	111.0 (19)
C2—C1—N1	120.32 (15)	H14C—C14—H14B	105 (3)
C6—C1—N1	119.48 (16)	C13—C14—H14A	114.9 (17)
C3—C2—C1	120.67 (18)	H14C—C14—H14A	106 (2)
C3—C2—H2	119.7	H14B—C14—H14A	107 (2)
C1—C2—H2	119.7	C20—C15—C16	120.46 (17)
C4—C3—C2	119.9 (2)	C20—C15—O2	123.22 (17)
C4—C3—H3	120.1	C16—C15—O2	116.30 (16)
C2—C3—H3	120.1	C15—C16—C17	119.01 (19)
C3—C4—C5	119.4 (2)	C15—C16—H16	120.5
C3—C4—H4	120.3	C17—C16—H16	120.5
C5—C4—H4	120.3	C18—C17—C16	121.9 (2)
C4—C5—C6	122.25 (19)	C18—C17—H17	119
C4—C5—H5	118.9	C16—C17—H17	119
C6—C5—H5	118.9	C17—C18—C19	117.39 (19)
C5—C6—C1	117.52 (18)	C17—C18—C21	121.3 (2)
C5—C6—C7	121.31 (18)	C19—C18—C21	121.3 (2)
C1—C6—C7	121.15 (17)	C18—C19—C20	122.0 (2)
C6—C7—H7A	109.5	C18—C19—H19	119
C6—C7—H7B	109.5	C20—C19—H19	119
H7A—C7—H7B	109.5	C15—C20—C19	119.2 (2)
C6—C7—H7C	109.5	C15—C20—H20	120.4
H7A—C7—H7C	109.5	C19—C20—H20	120.4
H7B—C7—H7C	109.5	C18—C21—H21A	109.5
C9—C8—C13	120.81 (18)	C18—C21—H21B	109.5
C9—C8—N2	120.30 (17)	H21A—C21—H21B	109.5
C13—C8—N2	118.87 (16)	C18—C21—H21C	109.5
C8—C9—C10	120.1 (2)	H21A—C21—H21C	109.5
C8—C9—H9	119.9	H21B—C21—H21C	109.5
C10—C9—H9	119.9	C1—N1—P1	123.77 (12)
C11—C10—C9	119.7 (2)	C1—N1—H1	120.6 (13)
C11—C10—H10	120.1	P1—N1—H1	115.4 (13)
C9—C10—H10	120.1	C8—N2—P1	127.71 (12)
C10—C11—C12	119.9 (2)	C8—N2—H21	113.0 (14)
C10—C11—H11	120.1	P1—N2—H21	119.2 (14)
C12—C11—H11	120.1	C15—O2—P1	124.67 (11)
C11—C12—C13	121.9 (2)	O1—P1—O2	113.30 (7)
C11—C12—H12	119	O1—P1—N1	118.95 (8)
C13—C12—H12	119	O2—P1—N1	96.87 (7)
C12—C13—C8	117.46 (19)	O1—P1—N2	109.57 (7)
C12—C13—C14	120.5 (2)	O2—P1—N2	110.16 (8)
C8—C13—C14	122.06 (18)	N1—P1—N2	107.23 (7)
C13—C14—H14C	112.0 (18)		
			
C6—C1—C2—C3	−1.1 (3)	C15—C16—C17—C18	−0.1 (3)
N1—C1—C2—C3	179.94 (17)	C16—C17—C18—C19	0.6 (3)
C1—C2—C3—C4	−0.7 (3)	C16—C17—C18—C21	−178.7 (2)
C2—C3—C4—C5	1.3 (4)	C17—C18—C19—C20	−0.8 (3)
C3—C4—C5—C6	−0.2 (4)	C21—C18—C19—C20	178.5 (2)
C4—C5—C6—C1	−1.5 (3)	C16—C15—C20—C19	0.0 (3)
C4—C5—C6—C7	176.6 (2)	O2—C15—C20—C19	−178.57 (19)
C2—C1—C6—C5	2.1 (3)	C18—C19—C20—C15	0.5 (3)
N1—C1—C6—C5	−178.90 (17)	C2—C1—N1—P1	39.1 (2)
C2—C1—C6—C7	−175.95 (18)	C6—C1—N1—P1	−139.85 (15)
N1—C1—C6—C7	3.0 (3)	C9—C8—N2—P1	45.5 (2)
C13—C8—C9—C10	−0.6 (3)	C13—C8—N2—P1	−135.66 (15)
N2—C8—C9—C10	178.22 (17)	C20—C15—O2—P1	−33.5 (2)
C8—C9—C10—C11	−1.8 (3)	C16—C15—O2—P1	147.94 (14)
C9—C10—C11—C12	2.0 (3)	C15—O2—P1—O1	62.76 (15)
C10—C11—C12—C13	0.2 (3)	C15—O2—P1—N1	−171.60 (13)
C11—C12—C13—C8	−2.4 (3)	C15—O2—P1—N2	−60.38 (15)
C11—C12—C13—C14	177.9 (2)	C1—N1—P1—O1	−75.73 (15)
C9—C8—C13—C12	2.6 (3)	C1—N1—P1—O2	162.81 (14)
N2—C8—C13—C12	−176.18 (16)	C1—N1—P1—N2	49.18 (16)
C9—C8—C13—C14	−177.7 (2)	C8—N2—P1—O1	169.91 (14)
N2—C8—C13—C14	3.5 (3)	C8—N2—P1—O2	−64.80 (17)
C20—C15—C16—C17	−0.2 (3)	C8—N2—P1—N1	39.51 (17)
O2—C15—C16—C17	178.44 (18)		

### Hydrogen-bond geometry (Å, °)

**Table:**

*D*—H···*A*	*D*—H	H···*A*	*D*···*A*	*D*—H···*A*
N1—H1···O1^i^	0.91 (2)	2.02 (2)	2.8963 (19)	161 (2)

Symmetry codes: (i) −*x*+1, *y*−1/2, −*z*+1/2.
